# Intrafollicular immature oocyte transfer (IFIOT): technical advances, biological insights and applications in reproductive biotechnologies

**DOI:** 10.1590/1984-3143-AR2026-0059

**Published:** 2026-08-20

**Authors:** José Felipe Warmling Sprícigo, José Eduardo Vieira Chaves, Margot Alves Nunes Dode

**Affiliations:** 1 Programa de Pós-Graduação em Ciência Animal – PPGCA, Universidade Federal de Goiás – UFG, Goiânia, GO, Brasil; 2 EMBRAPA Recursos Genéticos e Biotecnologias, Brasília, DF, Brasil

**Keywords:** follicle, injection, cumulus–oocyte complexes, embryo

## Abstract

Intrafollicular immature oocyte transfer (IFIOT) represents an intermediate approach between in vitro embryo production (IVP) and conventional in vivo–derived embryo systems, combining key advantages of both. Similar to IVP, it enables repeated use of donor females through ovum pick-up without gonadotropin-based superstimulation, while allowing oocyte maturation, fertilization and early embryonic development to occur under physiological in vivo conditions. The development of IFIOT has progressed from early experimental demonstrations of intrafollicular maturation to ultrasound-guided applications and, more recently, to the production of viable embryos and live offspring, confirming its biological feasibility. However, efficiency remains limited, with embryo recovery rates of 3–17%, reflecting constraints such as oocyte retention, follicular disruption and suboptimal injection conditions. Beyond embryo production, IFIOT provides a valuable model to investigate follicular function and oocyte competence, with emerging applications in oocyte cryopreservation, embryo production from prepubertal donors, and somatic cell nuclear transfer. Therefore, IFIOT should be viewed not only as an embryo production alternative, but as a physiological platform for in vivo oocyte maturation with broad applications in farm animals and wildlife species, while further technical refinement may enable its consolidation as an efficient system for embryo production.

## Introduction

Bovine embryo production is a key tool in modern livestock systems, enabling rapid dissemination of superior genetics and accelerating genetic gain (VanRaden, 2004; Crowe et al., 2021; Viana, 2024. Currently, it relies on two main approaches: in vivo systems, which provide a physiological environment and generate high-quality embryos, and in vitro embryo production (IVP), which offers greater flexibility and scalability (Hansen, 2024). However, both strategies have important limitations. In vivo systems are constrained by cost, variability, and the need for superstimulatory gonadotropin treatments (Bó and Mapletoft, 2014; Hasler, 2014), whereas IVP remains less efficient and consistently produces embryos with reduced developmental competence, largely due to its inability to replicate the physiological environment (Ealy et al., 2019; Hansen, 2024). These limitations are associated with alterations in lipid metabolism, mitochondrial organization, and gene expression (Crosier et al., 2000; Sudano et al., 2014; Urrego et al., 2014).

To overcome these limitations, intrafollicular immature oocyte transfer (IFIOT) has emerged as an intermediate approach between IVP and conventional in vivo systems, combining key advantages of both. Similar to IVP, IFIOT enables repeated use of donor females through ovum pick-up, increasing oocyte availability without the need for gonadotropin-based superstimulation (Kassens et al., 2015; Sprícigo et al., 2016). At the same time, unlike IVP, it allows oocyte maturation, fertilization, and early embryonic development to occur under physiological in vivo conditions, embryos generated withing the reproductive tract (Nicolás and Dode, 2024).

The development of IFIOT has progressed from early experimental demonstrations of intrafollicular oocyte maturation (Fleming et al., 1985b; Hinrichs and DiGiorgio, 1991; Bergfelt et al., 1998) to the establishment of ultrasound-guided techniques that enabled its practical application in cattle, using in vitro matured oocytes (Kassens et al., 2015). Subsequent studies demonstrated that transferred oocytes can complete maturation, be fertilized, and generate viable embryos and live offspring, confirming the biological feasibility of the technique (Spricigo and Dode, 2016; Sprícigo et al., 2016). Despite these advances, the efficiency of IFIOT remains limited, with embryo recovery rates typically ranging from 3 to 17% , reflecting both biological and technical constraints, including oocyte retention after injection, follicular disruption, synchronization of ovulation, and variability in oocyte competence and gamete transport (Nicolás and Dode, 2024).

Beyond its application as an embryo production system, IFIOT also provides a valuable experimental platform to investigate follicular function and the mechanisms regulating oocyte competence. In this context, its use for in vivo oocyte maturation has emerged as a complementary approach, particularly in challenging scenarios such as oocyte cryopreservation, somatic cell nuclear transfer, and the use of prepubertal donors, all of which are associated with reduced oocyte developmental competence (Chaves et al., 2025; Silva et al., 2026). Furthermore, IFIOT still holds significant potential to study and improve the competence of intrinsically compromised oocytes, such as those derived from aged females or high-producing dairy cows. In addition to cattle, IFIOT has been explored in other species, including sheep and horses, and may represent a promising alternative for assisted reproduction in wildlife species where in vitro systems remain suboptimal (Falchi et al., 2022) (Andino et al., 2019).

This review provides a comprehensive overview of IFIOT, addressing its technical evolution, biological foundations, current limitations, and emerging applications in reproductive biotechnologies.

## Historical evolution and technical advances in IFIOT

The origin of intrafollicular manipulation predates its recent application in cattle. Although live births have only been achieved more recently (Kassens et al., 2015; Spricigo and Dode, 2016), its foundations originate from experimental studies in the 1980s and 1990s, which were primarily focused on understanding the interactions between the oocyte and the follicular environment. Early in vitro studies using explanted porcine follicles demonstrated that intrafollicular maturation depends on COC integrity and gonadotropic stimulation, highlighting the role of follicular communication in meiotic regulation (Fleming et al., 1985a).

Subsequently, the technique was first applied under in vivo conditions. Fleming et al. (1985b) conducted one of the earliest intrafollicular transfer experiments in cattle and baboons, representing a key milestone in the development of this approach. At that time, donor females were previously superstimulated with FSH before ovariectomy for oocyte retrieval. The oocytes, in groups varying from 10 to 27, were transferred into the preovulatory follicle via laparotomy. The recovery of additional embryos following ovulation, and uterine flushing provided early evidence that IFIOT could support not only oocyte maturation, but also subsequent stages of the reproductive process in vivo.

In the following years, Hinrichs and DiGiorgio (1991) described, in mares, an IFIOT approach using a trocar and cannula system to access the preovulatory follicle. In that study, 20 transfers were performed and up to 80% of injected oocytes were recovered after 24 hours, showing cumulus expansion indicative of ongoing maturation. In addition, the recovery of more embryos than ovulations in some mares reinforced the hypothesis that transferred oocytes could complete maturation and contribute to embryo development in vivo.

An important methodological advance was the incorporation of transvaginal ultrasonography to access the recipient follicle. Carnevale and Ginther, (1993) described in mares an ultrasound guided intrafollicular transfer approach using an intravaginal probe, making the procedure less invasive and more applicable to assisted reproduction. In that study, the occurrence of twin pregnancies in two mares demonstrated that the transferred oocytes could support early embryonic development.

**Figure 1:**
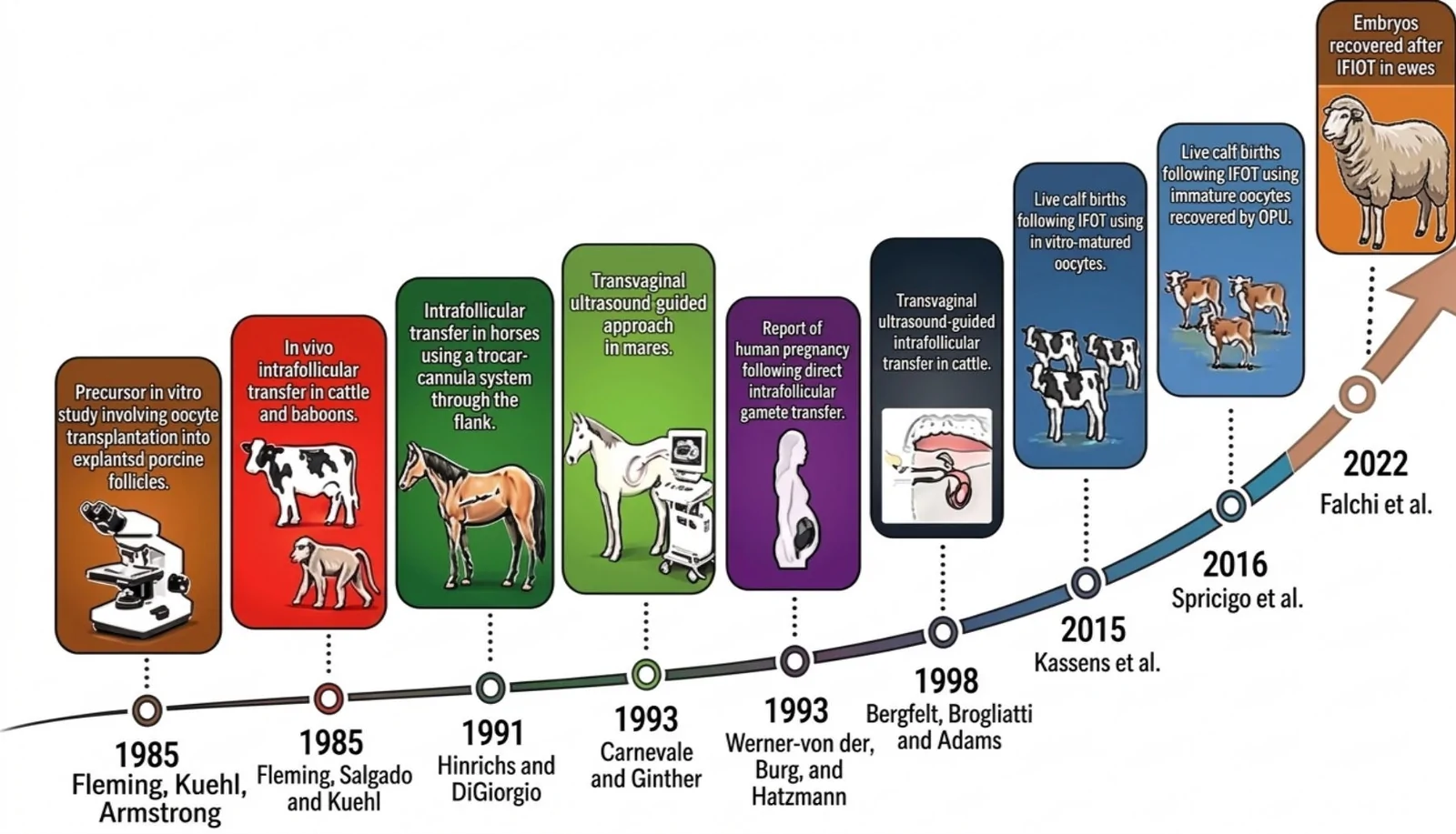
Timeline summarizing the main milestones in the development of intrafollicular oocyte transfer, including early experimental studies, technical advances, the first live births in cattle and embryos collected in ewes.

In cattle, the transvaginal ultrasound-guided approach was later described by Bergfelt et al. (1998), who termed the technique GRAFT (Gamete Recovery and Follicular Transfer). In this model, COCs were injected into the preovulatory follicle resulting in a 37% recovery rate, with approximately 50% of the recovered structures corresponding to early-stage embryos. These findings demonstrated the technical feasibility of the approach (Figure 1).

In parallel, the potential of the intrafollicular environment for immature oocyte transfer was also explored in humans. Werner-von der Burg et al., (1993) reported a pregnancy following direct intrafollicular transfer (DIFT), in which oocytes and spermatozoa were co-transferred into the follicle. Although not directly equivalent to IFIOT, this finding supported the concept that follicle can sustain fertilization and early embryonic development.

Despite these advances, offspring production was only achieved from 2010s onward (Table 1). Kassens et al. (2015) performed the intrafollicular oocyte transfer (IFOT) of in vitro matured oocytes in cattle,, reporting recovery rate of approximately 35% on d7 after AI, with about 9% developing to blastocysts, and the birth of the first live calf produced using this approach.

**Table 1:** Summary of intrafollicular oocyte transfer (IFOT) studies, including experimental conditions and embryo production outcomes (adapted from Nicolás and Dode, 2024).

Autor	Year	Specie	Obtaining COCs	COCs injected/animal	Collection day	Animals Collected (N°)	Animals yielding structures (N°)	Structures recovered	Structures recovery rate	Developed embryos recovered	Developed Embryos recovery rate
Fleming et al.	1985b	Bovine	Surgical	10-27	7 d. after injection	4	2	3	_	_	_
		Baboon	Post euthanasia	10-27	5 d. after injection	10	1	1	_	_	_
Hinrichs and DiGiorgio	1991	Equine	Abattoir	~ 15	7-11 d. after ovulation	15	4	_	_	16	_
Bergfelt et al.	1998	Bovine	OPU	5-6	2-3 d. after injection	7	5	16	37.2%	_	_
Deleuze et al.^a^	2009	Equine	OPU	2-10	10 d. after injection	10	4	_	_	6	12.8%
Deleuze et al.^b^	2009	Equine	OPU	3-14	10 d. after injection	8	2	_	_	3	5.5%
Kassens et al.	2015	Bovine	Abattoir	~ 60	7 d. after injection	28	26	583	35.2%	139	8.4%
Sprícigo et al.^a^	2016	Bovine	OPU	10-22	8 d. after injection	6	5	23	21.3%	6	5.5%
Sprícigo et al.^b^	2016	Bovine	Abattoir	~ 25	8 d. after injection	10	10	115	47.6%	31	12.9%
Hoelker et al.	2017	Bovine	Abattoir	~ 50	8 d. after injection	16	13	306	38.5%	147	17.3%
Andino et al.^a^	2019	Equine	OPU	2-9	9 d. after ovulation	13	0	_	_	0	_
Andino et al.^b^	2019	Equine	OPU	4-11	9 d. after ovulation	26	6	_	_	12	5.1%
Andrlikova et al.	2020	Bovine	Abattoir	15–24	7-8 d. after injection	16	6	71	22.5%	32	10.1%
Kolling et al.,	2022	Bovine	OPU	25	8 d. after injection	19	-	85	17.3%	19	3.9%
Falchi et al.	2022	Ovine	Abattoir	22-28	6 d. after mating	5	4	27	25.2%	13	12.1%
Nicolás et al. ^a^	2026	Bovine	Abattoir	25	8 d. after injection	9	9	58	21.7%	15	3.8%
Nicolás et al.^b^	2025	Bovine	Abattoir	50	8 d. after injection	8	8	78	19.8%	14	3.6%

a,b Indicate distinct experimental groups within the same study.

Subsequently, Sprícigo et al. (2016) further advanced the technique through two complementary experiments. In the first, immature oocytes recovered from slaughterhouse ovaries were directly transferred into preovulatory follicles of synchronized recipient cows. Following oocyte transfer, the cows were artificially inseminated, and embryo recovery was performed eight days later, resulting in an oocyte recovery rate of 47.6%, with 12.9% of the recovered structures developing into embryonic structures. In a second experiment, immature oocytes recovered by OPU from live donor cows were transferred into preovulatory follicles, followed by AI and embryo recovery. The recovered embryos were subsequently transferred to synchronized recipient females, resulting in four pregnancies after the transfer of 11 embryos. The births from these pregnancies were later reported by Sprícigo and Dode (2016). Together, these studies established the feasibility of producing live offspring through a completely in vivo embryo production system based on intrafollicular transfer of immature oocytes.

Recently, IFIOT in mares resulted in viable embryos after fertilization, although embryo recovery rates were inconsistent (Martinez de Andino et al., 2019). Similarly, experimental studies in sheep demonstrated ovulation and embryonic development following intrafollicular transfer (Falchi et al., 2022).

**Figure 2:**
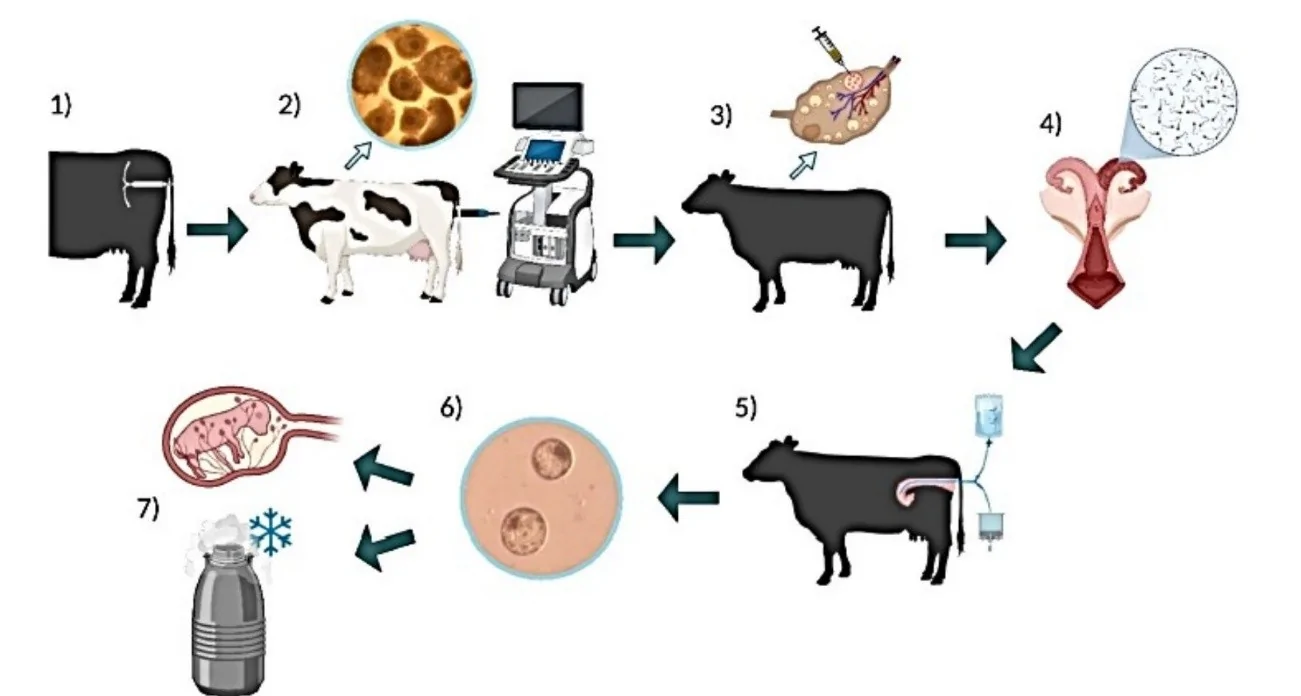
Overview of the IFIOT procedure: (1) hormonal synchronization; (2) COC collection; (3) intrafollicular injection; (4) artificial insemination; (5) embryo recovery; (6) embryo evaluation; and (7) embryo transfer or cryopreservation. Adapted from (Nicolás and Dode, 2024).

Currently, in cattle, the technique is predominantly performed using an OPU-based approach under controlled reproductive protocols. As presented in Figure 2, immature COCs are collected from donor females and injected into the dominant follicle of a synchronized recipient shortly before ovulation (~18-24h) for maturation. After insemination, fertilization and early embryonic development occurred in vivo, and embryos are recovered by uterine flushing approximately eight days after injection (Nicolás and Dode, 2024).

Although IFIOT is biologically feasible in cattle and other species, with studies reporting the production of viable offspring, its overall efficiency remains low. In cattle (Table 1), only 3–17% of injected oocytes develop into viable embryos (Nicolás and Dode, 2024). These limitations likely reflect multiple biological and technical constraints acting at different stages of the process.

## Biological determinants and technical limitations of IFIOT efficiency

Several biological determinants and technical limitations influence IFIOT efficiency. Key factors include hormonal control of ovulation and follicular competence, injection conditions, oocyte maturation, ovulation and gamete transport, as well as technical aspects such as injection volume, needle properties, and the medium used.

### Hormonal control of ovulation and follicular competence in IFIOT

As IFIOT depends on precise temporal coordination between oocyte injection, ovulation and insemination, ovulation synchronization is a key determinant of its efficiency (Kassens et al., 2015; Sprícigo et al., 2016; Hoelker et al., 2017). Early studies in *Bos taurus*, commonly employed GnRH-based protocols, similar to *Ovsynch*, to regulate follicular wave emergence and induce ovulation of a synchronized dominant follicle (Kassens et al., 2015; Hoelker et al., 2017).

Alternatively, progesterone–estradiol-based protocols have been widely adopted, particularly in studies conducted in Brazil (Sprícigo et al., 2016; Faria et al., 2021; Nicolás et al., 2025). In initial studies, oocyte injection was performed approximately 48 h after progesterone device removal, following estrus onset (Sprícigo et al., 2016). Subsequent studies shifted IFIOT to approximately 36 h after progesterone removal, prior to estrus and concomitant with GnRH administration, allowing more precise control of ovulation timing and ensuring that oocyte transfer occurred under a more consistent endocrine *milieu* during the periovulatory period (Faria et al., 2021; Nicolás et al., 2025).

Evidence suggests that differences in synchronization strategies may influence the recovery of structures following the procedure, likely through their effects on follicular development and the physiological status of the preovulatory follicle. Kolling et al., (2022), working with Nelore cows (*Bos indicus)*, reported similar recovery rate of structures and embryos on D7 after IFOT and AI, when using an estradiol-based protocol (13.6% and 3.9%) compared with an *Ovsynch* protocol (11.2% and 3.7%). Comparable recovery rates (approximately 12–13% of injected oocytes) have been reported in studies using progesterone–estradiol-based protocols (Nicolás et al., 2025), while procedural factors may additionally influence follicular dynamics and oocyte recovery (Faria et al., 2026). In this context, the effectiveness of IFIOT appears to depend not only on synchronization per se, but also on the ability of these protocols to generate a physiologically competent follicle at the time of oocyte transfer.

Follicle size is commonly used as an indirect indicator of follicular physiological and endocrine status, with follicles closer to ovulation providing more favorable conditions for oocyte competence. Kassens et al. (2015) reported higher embryo recovery rates in follicles measuring 13–14 mm; however, this relationship is breed-dependent. In *Bos taurus*, preovulatory follicles are typically ≥14 mm (Kassens et al., 2015; Hoelker et al., 2017), whereas in *Bos indicus*, follicles become responsive to LH at smaller diameters (>8 mm), with 10–12 mm follicles being considered functionally preovulatory (Sartori and Barros, 2011). Accordingly, Chaves et al. (2025) showed that IFIOT in preovulatory follicles of *Bos indicus* yields high MII rates in fresh oocytes (90.2%), comparable to those obtained with in vitro maturation (89.8%), despite smaller follicle size.

### Injection conditions

Technical aspects of the intrafollicular injection procedure can also influence IFIOT efficiency, including needle characteristics, injection volume, vehicle medium, number of injected oocytes and operator skill.

Needle characteristics represent an important technical factor influencing IFIOT outcomes, as they must allow the passage of intact COCs while minimizing mechanical damage to the follicular wall, which could lead to leakage of follicular contents and loss of oocytes. Previous studies conducted in cattle have used needles ranging from 24 G to 27 G (Bergfelt et al., 1998; Kassens et al., 2015; Sprícigo et al., 2016; Hoelker et al., 2017; Simões et al., 2021). Recent studies evaluating different needle types (27G spinal, 27G WTA, 27G gingival and 30G gingival) demonstrated that smaller internal diameters increased the risk of COC denudation and mechanical damage during injection (Nicolás et al., 2025). In particular, the 27G gingival and 30G needles were associated with higher denudation rates, whereas the 27G spinal needle, with a larger internal diameter, better preserved cumulus cell integrity and maintained high recovery rates. These findings highlight that needle selection is a critical factor affecting IFIOT efficiency.

Injection volume is another technical factor that may influence IFIOT outcomes by affecting the intrafollicular distribution of COCs. Early bovine studies commonly used relatively large volumes of vehicle medium, typically ranging from 60 to 200 μL (Kassens et al., 2015; Sprícigo et al., 2016; Hoelker et al., 2017; Andrlíková et al., 2020). Experimental evaluation demonstrated that, although small volumes (10–20 μL) are sufficient for oocyte injection in vitro, larger volumes improved the recovery of structures in vivo. For example, increasing the injection volume from approximately 10 μL to 60 μL improved the recovery rate of structures at Day 7 post-IA (43.4% vs. 16.9%), although embryo recovery rates remained unaffected.. These findings suggest that larger volumes may facilitate dispersion of COCs within the follicular antrum, increasing the likelihood of their release during ovulation, and, consequently, improving recovery efficiency. In contrast, studies in sheep have shown that smaller injection volumes (~5 μL containing 30 COCs), combined with intermediate needle diameters, can also result in high recovery rates while preserving cumulus cell integrity (Falchi et al., 2022). These differences likely reflect species-specific follicular characteristics and experimental conditions, indicating that optimal injection parameters may vary depending on the biological context.

The number of injected oocytes per follicle also varies considerably among studies, typically ranging from 5 to 60 COCs (Kassens et al., 2015; Sprícigo et al., 2016; Hoelker et al., 2017). Experimental comparisons using 25 or 50 injected COCs indicated that increasing the number of oocytes did not significantly affect the recovery of structures or embryos, suggesting that transferring larger numbers of COCs may be feasible without compromising the procedure (Nicolás et al., 2025). However, more recent evidence suggests that increasing the number of injected COCs may affect the follicular environment, as follicles injected with 50 COCs showed reduced antioxidant capacity and altered expression of genes related to oxidative stress and cellular response (SOD1, GPX4, NFE2L2, HSP70 and CASP3), despite no differences in cellular damage as assessed by cfDNA levels (Kussano et al., personal communication). Therefore, it is still unclear how many COCs can be injected without compromising the follicular environment.

Finally, the quality of the injection procedure itself may also influence the outcome of IFIOT. High-quality injections are characterized by accurate and stable deposition of COCs in the central region of the follicle with clear visualization of their entry into the follicular fluid, whereas poor-quality injections may involve peripheral deposition, vortex-like flow, multiple follicular punctures, or reduction in follicle diameter, indicating leakage of follicular contents (Faria et al., 2026). Experimental evidence indicates that the injection process itself can induce transient follicular regression and reductions in follicular diameter and volume, likely associated with partial loss of follicular fluid following needle penetration (Faria et al., 2026). Moreover, Nicolas et al. (2025) reported recovery rates of 24.7% and 16.9% following well-executed and poor-quality injections, respectively; however, these differences were not statistically significant. Nevertheless, minimizing follicular trauma remains an important procedural objective to preserve COCs within the follicular cavity..

An additional limitation of IFOT is the presence of a native oocyte within the recipient follicle, which may be fertilized and generate an embryo unrelated to the transferred oocytes. This possibility was reported by Kassens et al. (2015) and Spricigo and Dode (2016). In those studies, donor and recipient animals were from different breeds, allowing calf phenotypes to confirm the origin of the pregnancy. Therefore, the native oocyte represents a potential source of undesired embryos and may compromise the traceability of the technique. A possible strategy to overcome this limitation is the use of the donor as its own recipient, in which oocytes aspirated from follicles in one ovary are transferred into a preovulatory follicle in the contralateral ovary; however, this approach has shown limited efficiency in equine models (de Andino et al., 2019).

### Oocyte maturation

Oocyte maturation is a coordinated process involving nuclear progression and extensive cytoplasmic and metabolic remodeling over approximately 18–24 h (Ma, 2001). While nuclear maturation progresses to metaphase II (MII), developmental competence depends largely on cytoplasmic events, including organelle redistribution, mitochondrial reorganization, lipid metabolism and redox regulation (Hyttel et al., 1997; Ferreira et al., 2009). Under in vitro conditions premature meiotic resumption often leads to asynchrony between nuclear and cytoplasmic maturation (Ma, 2001).

In this regard IFIOT allows oocyte maturation within the physiological follicular environment, promoting a more synchronized progression of these events. Oocytes matured intrafollicularly reach MII rates comparable to in vitro systems but exhibit higher fertilization rates and improved embryo development (Silva et al., 2026), although prolonged exposure leads to oocyte aging and loss of competence (Simões et al., 2021).

Intrafollicular maturation also supports key cytoplasmic processes associated with competence, including homogeneous mitochondrial distribution, with organelles preferentially localized in central regions of the ooplasm and in close association with lipid droplets, reinforcing the existence of a coordinated metabolic organization during maturation (Chaves et al., 2025). In addition, oocytes matured in vitro show increased lipid accumulation and oxidative stress, whereas IFIOT maintains lipid content and redox balance closer to in vivo conditions (Faria et al., 2021; Chaves et al., 2025). Together, these findings reinforce that the intrafollicular environment supports a more physiological coupling between lipid metabolism and mitochondrial function, which is essential for the developmental competence. At the molecular level, transcript abundance of genes related to lipid metabolism, are largely preserved in oocytes matured via IFIOT compared with in vivo system, further supporting that the follicular environment preserves key metabolic pathways required for oocyte competence (Faria et al., 2021).

### Ovulation and gamete transport

During the preovulatory period, increasing estradiol concentrations induce the LH surge, which triggers final oocyte maturation and ovulation approximately 24–32 h later (Sartori and Barros, 2011). Following ovulation, the oocyte is captured by the fimbriae and transported to the oviduct, where fertilization occurs within a narrow temporal window (Gordon, 2003). The oviduct also provides a regulated environment that supports fertilization and early embryonic development (Aguilar and Reyley, 2005).

In the context of IFIOT, it is expected that these events occur in a physiological setting, as both the ovulatory follicle and the oviduct should be exposed to the same endocrine mileu and structural processes that support ovulation, fertilization, and the transport of gametes and early embryos. However, despite this expectation, efficiency remains limited, with recovery of structures after flushing ranging from 10 to 50% (Kassens et al., 2015; Hoelker et al., 2017; Nicolás and Dode, 2024). Evidence indicates that follicular dynamics are not impaired, as injected follicles continue to grow and ovulate (Faria et al., 2021, 2026), although a reduction in follicular diameter and volume after injection suggests mechanical disturbance and possible fluid loss (Faria et al., 2026). Consistent with this, preliminary observations (Kussano et al., personal communication) indicate that follicular puncture itself negatively affects follicular integrity, regardless of whether fluid or oocytes are injected, supporting the hypothesis that mechanical disruption may contribute to early oocyte loss.

Studies following IFIOT shows that approximately 40–60% of injected oocytes can be recovered prior to ovulation by OPU of the dominant follicle (Simões et al., 2021; Chaves et al., 2025), indicating that incomplete recovery likely reflects the partial retention of oocytes within the follicular environment. However, recovery of total structures on D7 is of a similar magnitude, generally differing by only 5–10%. Although these approaches are not directly comparable, this pattern suggests that a considerable proportion of oocytes may be lost shortly after injection, possibly associated with leakage of follicular fluid through the injection site, rather than exclusively during ovulation or transport. At the same time, incomplete oocyte release may also contribute to losses, as oocytes can remain trapped within follicular remnants or the corpus luteum (Carvalho et al., 2026). Together, these observations suggest that IFIOT efficiency is likely constrained by events occurring both immediately after injection and during periovulatory processes, although the relative contribution of each step remains to be fully elucidated.

## Applications of intrafollicular oocyte maturation for improving reproductive biotechnologies

As discussed above, IFIOT has been demonstrated to be biologically feasible: however, its efficiency remains limited, indicating that important biological and technical constraints still need to be overcome. IFIOT should therefore be viewed not only as an alternative embryo production strategy, but also as a valuable experimental model to investigate broader aspects of follicular function and the regulation of the intrafollicular environment.

By allowing the final stages of oocyte maturation to occur within a physiological environment, IFIOT may better support coordinated nuclear and cytoplasmic maturation compared to in vitro system. This approach is particularly relevant for addressing key limitations of IVP, with potential applications in improving the competence of cryopreserved oocytes, enhancing developmental potential in oocytes derived from prepubertal donors, and supporting cytoplasmic quality in advanced technologies such as somatic cell nuclear transfer. The following sections explore these applications and their implications as research avenues for improving reproductive biotechnologies

### Oocyte cryopreservation

Oocyte cryopreservation remains inefficient due to cryo-injuries, particularly in immature oocytes with high water and lipid content, which compromise cellular structures and metabolic competence (Katz-Jaffe et al., 2009; Sudano et al., 2011; Del Collado et al., 2017; Faria et al., 2021). In this context, in vivo maturation systems, such as IFIOT, could provide a more physiological environment that supports oocyte competence with positive effects demonstrated in fresh oocytes (Sprícigo et al., 2016; Faria et al., 2021; Chaves et al., 2025). However, the application of IFIOT to vitrified immature oocytes resulted in no recoverable embryos at uterine flushing on Day 7 after insemination, suggesting that cryopreservation at the immature stage severely compromised embryo development (Sprícigo et al., 2016), and oocytes cryopreserved at the immature stage and subsequently matured via IFIOT showed markedly lower in vitro embryo production rates compared to non-cryopreserved controls (Chaves et al., 2025). These findings indicate that IFIOT is unable to reverse severe cryo-injury. Therefore, an alternative strategy may be to perform oocyte maturation via IFIOT prior to vitrification, allowing oocytes to reach MII stage under physiological conditions, followed by in vitro embryo production with potentially improved developmental competence.

### Potential improvements in oocyte quality for somatic cell nuclear transfer (SCNT)

In SCNT, the developmental success of the reconstructed embryo relies primarily on the cytoplasmic competence of the recipient oocyte, which must support nuclear reprogramming and early embryonic development (Yang et al., 2007; Meng et al., 2021). Therefore, improving the quality of the oocyte cytoplasm is a key requirement for making cloning more efficient and viable. However, in vitro matured oocytes often exhibit metabolic and cytoplasmic deficiencies that impair development, whereas oocytes matured via IFIOT have shown improved quality, including better cytoplasmic characteristics and reduced lipid accumulation (Faria et al., 2021; Chaves et al., 2025). This improved cytoplasmic competence is particularly relevant and could improve cloning efficiency. Preliminary data (Chaves et al., personal communication) indicate that oocytes matured via IFIOT may increase fusion rates and show a tendency toward higher embryonic development rates compared to in vitro–matured oocytes. Although preliminary, these findings support the hypothesis that physiological maturation systems such as IFIOT can enhance oocyte quality and improve overall outcomes in SCNT.

### Use in prepubertal donors with reduced oocyte competence

In prepubertal donors, oocyte competence is intrinsically limited by cytoplasmic immaturity, altered lipid metabolism, and suboptimal mitochondrial function (Gandolfi et al., 1998; Sturmey et al., 2009; Lodde et al., 2013). Consistent with this, oocytes matured via IFIOT exhibited reduced lipid accumulation and lower mitochondrial activity, indicating a more regulated metabolic state (Silva et al., personal communication). These changes were associated with numerically greater blastocyst rates in prepubertal calves subjected to IFIOT (14.1%) than in those matured exclusively in vitro (7.6%) (Silva et al., 2026). This approach may also be particularly relevant in other contexts of reduced oocyte competence, such as in senescent cows or high-producing dairy females, where metabolic stress and altered follicular environments can impair oocyte quality.

## Conclusions and future perspectives

Intrafollicular transfer of immature oocytes (IFIOT) has evolved into a versatile tool in reproductive biotechnologies, extending beyond its initial proposal as an alternative embryo production system. In addition to its practical applications, the technique has become a valuable experimental model for studying oocyte maturation and follicular dynamics, providing insights into how the intrafollicular environment regulates cytoplasmic competence and developmental potential. Evidence from recent studies indicates that IFIOT can improve oocyte quality and be successfully associated with other biotechnologies, particularly under conditions where oocyte competence is compromised.

Despite these advances, the efficiency of IFIOT remains limited, and further optimization is required to expand its practical application. Future research should focus on improving oocyte recovery, refining technical procedures, and integrating IFIOT with in vitro embryo production systems. Moreover, the technique holds strong potential in contexts where conventional in vitro maturation systems are not well established, such as in wildlife species or interspecies applications. In this sense, IFIOT may serve as a physiological platform for oocyte maturation across species, including the potential use of bovine follicles to support the maturation of oocytes from other species, such as buffalo. Overall, although still under development, IFIOT represents a promising and expanding field with the potential to drive important advances in reproductive biotechnologies.

## Data Availability

No research data was used.
